# Normative ratings for the Kitchen and Food Sounds (KFS) database

**DOI:** 10.3758/s13428-024-02402-7

**Published:** 2024-03-28

**Authors:** Marília Prada, David Guedes, Margarida Vaz Garrido, Magda Saraiva

**Affiliations:** 1 Iscte - Instituto Universitário de Lisboa, Av. das Forças Armadas, 1649-026 Lisboa, Portugal; 2 William James Center for Research, Ispa-Instituto Universitário, Lisboa, Portugal

**Keywords:** Food sounds, Cooking sounds, Eating/drinking sounds, Sound ratings, Sound identification

## Abstract

Sounds are important sensory cues for food perception and acceptance. We developed and validated a large-scale database of kitchen and food sounds (180 stimuli) capturing different stages of preparing, cooking, serving, and/or consuming foods and beverages and sounds of packaging, kitchen utensils, and appliances. Each sound was evaluated across nine subjective evaluative dimensions (random order), including stimuli-related properties (e.g., valence, arousal) and food-related items (e.g., healthfulness, appetizingness) by a subsample of 51 to 64 participants (*Mdn* = 54; *N* = 332; 69.6% women, *M*_age_ = 27.46 years, *SD* = 10.20). Participants also identified each sound and rated how confident they were in such identification. Results show that, overall, participants could correctly identify the sound or at least recognize the general sound categories. The stimuli of the KFS database varied across different levels (low, moderate, high) of the evaluative dimensions under analysis, indicating good adequacy to a broad range of research purposes. The correlation analysis showed a high degree of association between evaluative dimensions. The sociodemographic characteristics of the sample had a limited influence on the stimuli evaluation. Still, some aspects related to food and cooking were associated with how the sounds are evaluated, suggesting that participants’ proficiency in the kitchen should be considered when planning studies with food sounds. Given its broad range of stimulus categories and evaluative dimensions, the KFS database (freely available at OSF) is suitable for different research domains, from fundamental (e.g., cognitive psychology, basic sensory science) to more applied research (e.g., marketing, consumer science).

## Introduction

Sound is an integral part of the eating experience. Much of our enjoyment of foods and drinks comes from sonic cues, such as anticipatory sounds, like the popping of popcorn, or those resulting from our direct interaction with food products, like the sound of biting a crunchy apple (for reviews, see Spence, 2015, 2017; Zampini & Spence, 2010).

In the advertisement of food and drink products, sound cues are often convened to create more engaging experiences (Knöferle & Spence, 2021). One illustrative example is Coca-Cola’s “Taste the Feeling” campaign, which called attention to the sonic experience surrounding the product, from bottle cap noises to the ice cube clicking, but also the fizzing sound of effervescence and the voicing of consumer satisfaction “aah” (Graakjær, 2021; Unger, 2017). This multisensory approach to marketing and advertising is backed by scientific research evidencing the influence of sonic cues on the perception of foods and drinks (Spence, 2017). For example, Zampini and Spence (2005) found that participants expected sparkling water to be significantly more carbonated when they listened to its fizzing sound amplified or when just the high-frequency components (2–20 kHz) were augmented (compared to when the sound was unaltered or attenuated). Although this effect did not hold for the actual oral experience, such findings may still hint at how sounds may aid in setting up the right expectations regarding the sensory attributes of drinks.

In food products, attributes such as crispness, crunchiness, smoothness, or hardness are also intimately associated with the auditory modality. One pivotal study found that manipulating the loudness and frequency of the sound feedback of eating a potato chip resulted in higher crispness and freshness ratings (Zampini & Spence, 2004). More recent research suggests that mastication sounds may also improve the perception of softened foods. Endo et al. (2016) had healthy adults taste nursing care foods while listening to prerecorded chewing sounds synchronized with their masticatory movements. The authors found that despite the evaluated foods being intrinsically soft, participants perceived them as having a firmer texture when eating was accompanied by chewing sounds.

Besides having an immediate influence on the perception of sensory attributes, sonic cues may also contribute to building more positive expectations toward products (Wang & Spence, 2019). For instance, listening to the sound of opening a bottle of wine with a cork (vs. screwcap) may lead to higher expectations of quality, as well as to a more favorable affective response (Spence & Wang, 2017). Similarly, it seems that the sounds of opening a bottle of beer (vs. can) and pouring (vs. opening) are liked more by consumers and foster the perception of the product as more “premium.” Interestingly, presenting pouring sounds in an e-commerce context may contribute to a more favorable affective response to a nonalcoholic beer, suggesting that sonic cues may have useful commercial applications in the digital world (Rodríguez et al., 2021). Researchers have also recently begun to explore the potential of sound for enriching the multisensory virtual reality experiences in food advertising (e.g., Brengman et al., 2022). However, audition remains a poorly explored sensory modality in virtual reality compared with the visual domain (Wang et al., 2021).

In the “food porn” era, foods are being depicted according to an increasingly appealing and suggestive aesthetic (Taylor & Keating, 2018). In this context, multisensory cues may become highly relevant to effectively communicate how foods taste, smell, feel, and sound. There is also growing popularity of ASMR (short for autonomous sensory meridian response) content in social media platforms and advertising where food is a ubiquitous object (for a review, see Spence, 2020). Some common sounds in ASMR content may include unwrapping candy, cracking pieces of chocolate, sizzling steaks, or consumption sounds such as biting, chewing, or slurping. This may suggest the importance of sound for the eating experience and that these food-related sounds may constitute a broader and more diverse category of stimuli than what has been recognized thus far. These sounds can precede or occur during consumption. The former may include the sounds of preparing foods and drinks (e.g., stirring, frying) as well as those associated with packaging (e.g., uncorking a wine bottle, popping open a bag of chips) or with the use of kitchen appliances (e.g., microwave oven, electric mixer). The latter category most notably includes those sounds resulting from human–food interaction, such as the consumption sounds (e.g., masticating) that serve as relevant information regarding the sensory properties of foods and drinks (e.g., crispness, crunchiness, carbonated; Spence, 2016).

### Norming studies of food-related stimuli

Although sound is undoubtedly a relevant sensory modality for research with food, there is a significant lack of validated auditory stimuli for this domain of study. Overall, norming studies in the auditory domain are still scarce relative to other senses, such as visual stimuli (Gerdes et al., 2014; Prada et al., 2016; Rodrigues et al., 2018; Souza et al., 2020, 2021; Yang et al., 2018). This scenario also applies to food-related stimuli, where stimulus sets for the visual modality thrive. For pictures, there are several hundreds of validated stimuli from datasets such as *food-pics* (Blechert et al., 2019), the FoodCast research image database (FRIDa, Foroni et al., 2013), the Open Library of Affective Foods (OLAF, Miccoli et al., 2016), Standardized Food Images (SFI, Charbonnier et al., 2016), or the CROss-CUltural Food Image Database (CROCUFID, Toet et al., 2019). Together, these datasets allow researchers to select the most appropriate visual materials to suit their research needs. The availability of norming data allows for higher experimental reproducibility and comparability across studies (Lepping et al., 2016; Shafiro & Gygi, 2004), as well as permitting the manipulation of attributes of interest (e.g., Rodríguez-Martín & Meule, 2015). To date, this has remained an important challenge for food research with auditory stimuli, given that the availability of validated food-related sounds pales in comparison to what may be found for food images.

Still, it is possible to find food-related sounds dispersed over the existing databases of everyday sounds. For instance, among a broad range of stimulus categories (e.g., people, nature, transports), the International Affective Digitized Sounds (IADS-2, Bradley & Lang, 2007; IADS-E, Yang et al., 2018) also included food-related sounds such as eating noodles, pouring water, chewing, or a fizzing soda. These stimuli were rated in both affective (e.g., valence, arousal) and emotional dimensions (e.g., happiness, fear; only in the IADS-E). The Emo-Soundscapes (Fan et al., 2017) also include human sounds associated with eating and drinking (e.g., gulping, chewing), as well as mechanical sounds that include kitchen appliances (e.g., coffee machine), evaluated in affective dimensions (valence, arousal). Another database, the Norms for Environmental Sound Stimuli (NESSTI, Hocking et al., 2013), provides subjective ratings for affective (pleasure, arousal) and cognitive dimensions (e.g., representativeness, imageability) for environmental sounds. Among the various natural and man-made sounds, we may find examples of household items (e.g., cutlery, dishes) as well as kitchen appliances (e.g., toaster). The Taste & Affect Music Database (Guedes, Prada, Garrido, et al., 2023) also provides rating norms for stimuli to be used in food research. This set of 100 instrumental soundtracks was evaluated for basic taste correspondences such as sweetness, bitterness, saltiness, and sourness.

Overall, it seems that although seen as a relevant category of auditory stimuli, only a small number of food-related sounds have been validated to date. Importantly, these stimuli are currently scattered across different databases of more general sounds, with inconsistent approaches in terms of acoustic qualities and subjective rating dimensions. The current study aimed to overcome these limitations by (1) developing the first set of original food-related sounds encompassing a significant diversity of stimulus categories and (2) obtaining norming data for subjective dimensions that are relevant to the specific needs of food science research. To accomplish these goals, we recorded 180 sounds related to food and cooking. The sounds were evaluated in affective (i.e., valence, arousal) and food-related dimensions (i.e., healthfulness, appetizingness, association to sweetness, and association to savoriness) selected with the goal of facilitating cross-comparison with other databases of food-related (and non-food-related) stimuli. Moreover, we also asked participants to identify each sound and how confident they were in such identification, and to rate their familiarity with the stimulus.

## Method

### Participants

A sample of 332 respondents (69.6% women, 29.8% men, and 0.6% non-binary) aged 18 to 67 years (*M*_age_ = 27.46 years, *SD* = 10.20) volunteered to participate in a web survey. Participants were recruited via email, social media, and an online panel (35.8%, Clickworker). University students made up 52.7% of the sample, and 41% were active workers (with only 4.5% reporting working in food-related areas, such as hospitality or nutrition), with either secondary (49.4%) or higher education (47.6%). On average, participants’ households included three people (*M* = 3.25, *SD* = 1.53), with 36.7% having at least one child. Most participants (95.8%) were Portuguese nationals or from Portuguese-speaking countries (e.g., Brazil, Cabo Verde, 3.6%), and all reported having normal audition at the time of the study. Overall, participants reported a high interest in food and nutrition (*M* = 5.07, *SD* = 1.55, CI 95% [4.90,5.23]).

### Materials

The recording conditions aimed to replicate the context where most people usually experience food-related sounds, namely a domestic kitchen. Still, several fabric panels were placed to promote acoustic isolation and minimize internal and external background noise (e.g., echo, traffic). We used a portable recorder (Zoom Handy digital audio recorder) that includes a built-in stereo mic (unidirectional condenser, 90° XY stereo format; maximum sound pressure level: 120 dB SPL; gain: -∞ dB to +9 dB; rated input level: -∞ dB to −39 dBm; rated output level: 20 mW + 20 mW into 32 Ω load).

**Fig. 1:**
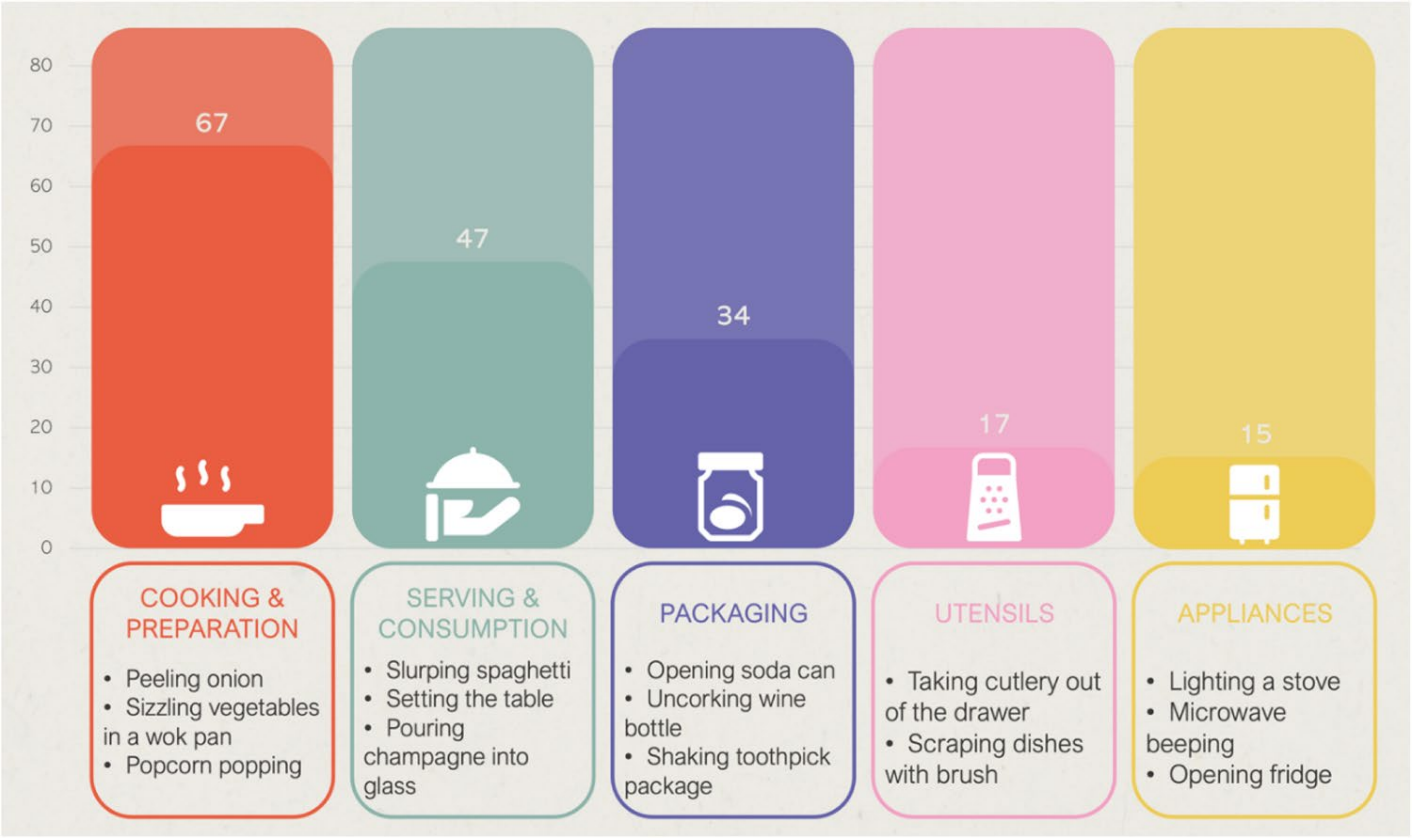
Sound categories (relative frequency and examples)

Our primary goal was to capture a comprehensive range of food-related sounds. Specifically, we asked a nonprofessional cook to perform multiple stages of food preparation, cooking, and even consumption. As in other normative studies including sound recording (e.g., Lima et al., 2013), this person was instructed to cook as they usually do. We also recorded sounds resulting from the manipulation of different types of food packaging, kitchen utensils, and kitchen appliances. Figure 1 illustrates the sounds recorded across these categories.

The resulting sound clips were prescreened by three researchers, and all that presented interference (e.g., the humming of the refrigerator compressor in the background) were excluded. The final set included 180 sounds (.mp4) edited so that all files had a 10-second duration. The sound files are freely available on the Open Science Framework (OSF).

### Measures and procedure

The study was approved by the ethics committee of Iscte–Instituto Universitário de Lisboa (Approval #117/2020). Participants were invited to collaborate on a survey (hosted on Qualtrics) exploring how people perceive different sounds. Specifically, they were asked to rate food-related sounds (e.g., food being prepared or consumed) across several dimensions. Instructions also emphasized the need to answer the survey in a quiet place using a computer and headphones.

Participants were also asked to confirm that they did not suffer from any permanent or transient hearing impairment at the time of the study that could impact their performance. The informed consent also included the expected duration of the study (about 25 minutes) and its compliance with the norms of ethical conduct in research (e.g., anonymity and confidentiality, voluntary nature of the participation, and the ability to withdraw from the study at any time). After agreeing with the terms of the informed consent, participants answered sociodemographic questions (e.g., gender, age, nationality, occupation).

Next, participants received detailed instructions about the task, namely the seven-point rating scales and the open-ended identification question (see Table 1). Participants were asked to provide subjective ratings for 30 sound clips randomly selected from the pool of 180 stimuli. After listening to the sound clip, participants rated each sound on nine evaluative dimensions presented in random order. Finally, they were asked to identify the sound (or simply indicate “*I don’t know*”), as well as their confidence in such identification.

**Table 1:** Subjective rating dimensions, instructions, and item scales

Dimension	This sound is… [instructions/scale anchors]
Valence	1 = *Not at all pleasant*; 7 = *Very pleasant*
Familiarity	1 = *Not at all familiar*; 7 = *Very familiar*
Intensity	1 = *Not at all intense*; 7 = *Very intense*
Healthfulness	1 = *Not at all healthful*; 7 = *Very healthful*
Appetizingness	1 = *Not at all appetizing*; 7 = *Very appetizing*
Arousal	1 = *Not at all arousing*; 7 = *Very arousing*
Associated with something sweet	1 = *Not at all sweet*; 7 = *Very sweet*
Associated with something savory	1 = *Not at all savory*; 7 = *Very savory*
Identification	Please identify the sound [open-ended response]
Confidence in the identification	1 = *Low confidence*; 7 = *High confidence*

The rating dimensions presented in Table 1 include more general affective variables, such as valence and intensity, as well as dimensions more closely related to the topic of food and eating. The choice of valence and intensity rests on the extensive body of research on the two-dimensional organization of affect (Yik et al., 1999, 2023). These dimensions are ubiquitous in existing datasets of auditory stimuli, regardless of their type (e.g., natural sounds—Bradley & Lang, 2007; Yang et al., 2018; vocalizations—Belin et al., 2008; Lassalle et al., 2019; Parsons et al., 2014; or music—Belfi & Kacirek, 2021; Imbir & Golab, 2017; Lepping et al., 2016; Song et al., 2016; Vieillard et al., 2008), as well as in food-related visual stimuli (e.g., Blechert et al., 2014, 2019; Foroni et al., 2013; Miccoli et al., 2016, Toet et al., 2019). Familiarity is another dimension of interest for auditory stimuli, not only for its putative influence on liking (Witvliet & Vrana, 2007) but also for its likely association with stimulus identification. In everyday situations, sound identification is significantly improved by the integration of multisensory cues (e.g., sound and image; Özcan & van Egmond, 2009). In the absence of appropriate contextual cues, the task of sound identification becomes increasingly challenging (e.g., a sound recording of a sizzling steak might sound strangely like a heavily rainy night). Although participants in this study were aware of a general context (food sounds), they still lacked complementary sensory cues for their evaluation task. Thus, in a task of this nature, the interpretation of subjective ratings depends on the degree of recognizability.

Finally, food-related dimensions included healthfulness and appetizingness, as well as associations with broad taste/flavor categories (sweet and savory). The former two dimensions allude to pivotal motivations for food consumption that refer to the rewarding or pleasurable aspects of eating, on the one hand, and longer-term concerns over fulfilling nutritional needs or contributing to improving health and fitness on the other (Renner et al., 2012). Unsurprisingly, these are two dimensions of interest in food-related visual stimuli as well, namely, in the form of healthiness and/or energy density metrics (Blechert et al., 2019; Charbonnier et al., 2016; Foroni et al., 2013; Toet et al., 2019) and a diverse set of hedonic or measures of hedonic reaction, such as palatability (Blechert et al., 2014, 2019), food craving (Miccoli et al., 2016), or desire to eat (Toet et al., 2019).

Previous studies show that sounds can also be associated with different taste/flavor dimensions (Guedes, Garrido, et al., 2023; Guedes, Prada, Garrido, et al., 2023). Although there are numerous possible taste and flavor descriptors for sounds, for the sake of parsimony, we opted to validate the two broader categories of sweet and savory food types (e.g., Blechert et al., 2014).

After evaluating the stimuli set, we asked participants to respond to measures characterizing their cooking experience (adapted from Kowalkowska et al., 2018): overall self-rating of cooking skills (“How do you evaluate your cooking skills?” 1 = *Poor* to 7 = *Excellent*); cooking frequency (“How frequently do you cook?” 1 = *Rarely* to 7 = *Frequently*); confidence (“How confident are you in your cooking skills?” 1 = *Not at all confident* to 7 = *Very confident*); and liking (“How much do you like cooking?” 1 = *I don’t like it at all,* 7 = *I like it a lot*). We also asked participants to respond to the Portuguese adaptation of the Cooking Skills Scale (CSS, Kowalkowska et al., 2018), which comprises seven items (e.g., “*I consider my cooking skills as sufficient*”; “*I am able to prepare a hot meal without a recipe*”; 1 = *Totally disagree* to 7 = *Totally agree*, Cronbach’s alpha = .861). Finally, we asked participants to indicate their overall interest in food and nutrition (1 = *Not at all interested* to 7 = *Very interested).* In the end, participants were thanked and debriefed.

### Data analytic plan

The complete normative data for the 180 stimuli on the nine evaluative dimensions, along with the descriptions for each sound (and respective confidence in the identification), are provided at OSF. Next, we present the following results: (a) preliminary analysis (e.g., outlier detection); (b) sound identification; (c) summary of the subjective rating norms for each dimension (i.e., percentage of sounds categorized as low, moderate, or high in a given dimension); (d) analyses of the impact of sound category across evaluative dimensions (multivariate analysis of variance [MANOVA], with sound category as the between-subjects variable); (e) correlations between evaluative dimensions; and (f) influence of individual characteristics (e.g., gender, age, cooking frequency) on overall ratings.

## Results

### Preliminary analysis

Only completed surveys (*N* = 332) were retained for the analysis. Therefore, no missing data were observed. Values situated 2.5 standard deviations above or below the mean evaluation of each stimulus were considered outliers (0.57%). As we did not detect evidence of systematic or random responses (e.g., consistent use of a single point of the scale), no participants were excluded.

We tested the consistency of participants’ ratings in each dimension by comparing two subsamples of equal size (*n* = 166) randomly selected from the main sample. No significant differences between the subsamples emerged (all *p* ≥ .185). Moreover, ratings across the nine evaluative dimensions were reliable (Cronbach’s alpha = .893; Spearman–Brown split-half reliability = .903).

### Sounds identification

Two coders independently evaluated participants’ responses to the sound identification task according to a four-level scheme. The highest score (level 1) was attributed to correct or nearly correct responses. For example, in stimulus 5 (preparing instant coffee), responses such as “pouring coffee” or “serving tea” were considered near correct and, as such, evaluated in the first level. Level 2 included all responses that correctly identified the sound as being associated with any of the categories (e.g., cooking/preparation, serving/consumption). The two remaining levels included responses suggesting that participants completely misattributed the origins of sounds (level 3) or reported being unable to identify the sound (level 4). For example, in stimulus 1 (microwave beeping), responses like “a truck parking/reversing” were scored as level 3, whereas responses like “I don’t know” or “I’m unable to identify” were scored as level 4. Scoring conflicts were resolved through team discussion.

**Fig. 2:**
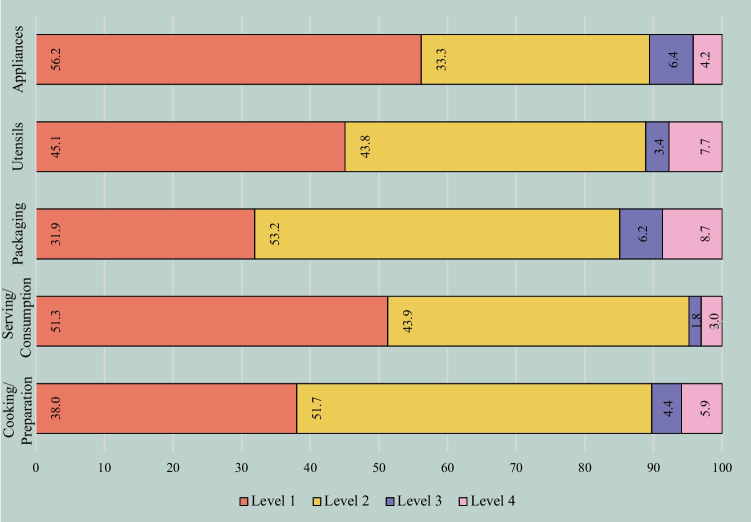
Response categories (relative frequency) per sound category. *Note*. Level 1 = correct (or near correct) identification; level 2 = incorrect identification but correct categorization as kitchen/food sound; level 3 = incorrect identification and categorization as kitchen/food sound; level 4 = no identification

Appendix 2 presents the identification findings (i.e.,% of each category, OSF) alongside the actual identification of each sound. Overall, participants accurately identified the sounds (*M*_Level1_ = 42.51%) or indicated one of the sound categories (*M*_Level2_ = 47.7%). Gross misattributions or “I don’t know” responses were infrequent (*M*_Level3_ = 4.1% and *M*_Level4_ = 4.7%, respectively). Figure 2 presents the overall distribution of response categories according to the type of sound.

The sound categories with the highest proportion of correct identifications (i.e., level 1) were Appliances and Serving/Consumption (both above 50%). Utensils sounds also obtained a high proportion of correct identification responses, followed by Cooking/Preparation and, finally, Packaging. Still, as shown in Table 2, all categories show a wide range in terms of accuracy of sound identification.

**Table 2:** Correct identification (mean relative frequency; minimum and maximum of level 1 responses) per sound category

Sound category	*M* %	Min %	Min Sound description	Max %	Max Sound description
Cooking/Preparation	38.0	1.9	De-seeding pomegranate (S174)	90.7	Cutting and chopping onion (S27)
Serving/Consumption	51.3	0.0	Biting ice cubes (S173)	96.2	Drinking juice from a straw (S147)
Packaging	31.9	2.0	Unwrapping and breaking chocolate bar (S105)	86.5	Opening can of soda (S91)
Utensils	45.1	7.8	Detaching paper towel (S22)	96.6	Taking cutlery out of the drawer (S121)
Appliances	56.2	13.7	Beeping fridge (S111)	85.2	Lighting a stove (S33)

### Subjective rating norms

**Fig. 3:**
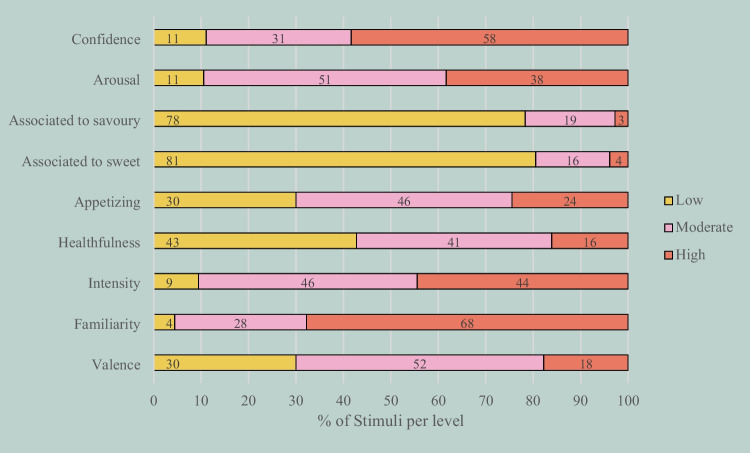
Distribution of items across dimension levels (low, moderate, high). *Note.* A stimulus was categorized as moderate on a given dimension if the confidence interval included the rating scale's midpoint. If the upper bound was lower than the scale's midpoint, the stimulus was considered low on that dimension, and if the lower bound was higher than the midpoint, the stimulus was considered high

Data were analyzed by sound to obtain the subjective rating norms. Each sound was evaluated by a minimum of 51 and a maximum of 64 participants (*Mdn* = 54). Appendix 1 presents the descriptive statistics (i.e., means, standard deviations, and confidence intervals on each dimension) per stimulus (available at OSF). To provide an overview of the database, based on the descriptive statistics (Table 3), we categorized the sounds as low, moderate, or high in each dimension (for a similar procedure, see Guedes, Prada, Garrido, et al., 2023; Prada et al., 2016; Rodrigues et al., 2018) and present the frequencies of each level in Fig. 3.

**Table 3:** Descriptive statistics per evaluative dimension

Dimension	Min	Max	*M*	*SD*	95% CI	
					LB	UB
Valence	1.07	6.43	3.87	0.86	3.78	3.97
Familiarity	2.00	6.90	4.92	0.78	4.84	5.01
Intensity	1.63	6.53	4.31	0.84	4.22	4.40
Healthfulness	1.13	5.33	3.80	0.69	3.73	3.87
Appetizingness	1.03	6.13	3.87	0.83	3.78	3.96
Association with something sweet	1.00	4.77	2.98	0.82	2.90	3.07
Association with something savory	1.07	4.93	3.02	0.82	2.93	3.11
Arousal	1.00	6.47	4.23	0.89	4.13	4.32
Confidence in the identification	1.37	6.53	4.65	0.88	4.56	4.75

Most stimuli were considered highly familiar (68%), moderately positive (52%), arousing (51%), and appetizing (46%). Regarding healthfulness, the distribution of sounds rated as low or moderate was similar (i.e., 43 and 41%, respectively). For intensity, roughly the same number of sounds were rated as moderate or high (i.e., 46 and 44%, respectively). Most sounds were rated as low concerning their association with sweet (81%) or savory foods (78%). Finally, most sounds were identified with high confidence (58%).

### Impact of sound category across evaluative dimensions

As shown in Table 4, the sound category significantly impacted ratings across all evaluative dimensions, all *p < .*001. Sounds in the Utensils category obtained the lowest scores in most dimensions—valence (but not different from Appliances, *p* = .460, all other *p ≤ *.006), intensity (but not different from Packaging and Cooking/Preparation, *p* ≥ .082, all other *p ≤ *.008), appetizingness (all *p* < .001), association to sweet (but not different from Cooking/Preparation, *p = *.267) and savory (but not different from Appliances and Cooking/Preparation, *p* ≥ .509, all other *p* < .001), and arousal (but not different from Utensils, *p* = .059, all other *p* < .001). Still, packaging sounds were rated as the least familiar (all *p* ≤ .011) and healthful (all *p < *.001), also obtaining the lowest confidence ratings (but not different from Utensils and Cooking/Preparation, *p* ≥ .060, all other *p* < .001).

**Table 4:** Evaluations (*M*, *SD*) per sound category

	App.		U		Pack.		S/C		C/P		Category effect		
	*M*	*SD*	*M*	*SD*	*M*	*SD*	*M*	*SD*	*M*	*SD*	*F*(4,1623)	*p*	*np*2
1. Valence	3.37	1.44	3.19	1.22	3.69	1.06	4.11	1.11	4.08	1.02	40.34	< .001	.090
2. Familiarity	5.25	1.35	4.79	1.39	4.50	1.10	5.33	0.91	4.83	0.91	29.90	< .001	.069
3. Intensity	4.70	1.26	3.99	1.26	4.11	1.10	4.59	0.97	4.22	0.92	24.63	< .001	.057
4. Healthfulness	3.84	1.26	3.41	1.10	3.35	0.89	3.77	0.86	4.15	0.84	35.21	< .001	.080
5. Appetizingness	3.60	1.44	2.89	1.29	3.55	1.11	4.34	1.05	4.01	0.97	69.74	< .001	.147
6. Associated with sweet	2.95	1.31	2.58	1.26	3.01	1.09	3.41	0.97	2.77	0.90	25.23	< .001	.059
7. Associated with savory	2.90	1.28	2.73	1.24	2.82	1.07	3.15	0.94	3.14	0.94	9.62	< .001	.023
8. Arousal	4.40	1.38	3.74	1.28	3.99	1.07	4.51	1.02	4.21	0.95	23.91	< .001	.056
9. Confidence	5.12	1.41	4.49	1.51	4.26	1.24	5.07	1.01	4.52	0.99	30.74	< .001	.070

*App* Appliances, *U* Utensils, *Pack* Packaging, *S/C* Serving/Consumption, *C/P* Cooking/Preparation

In contrast, sounds from the Serving/Consumption category obtained the highest scores in valence (but not different from Cooking/Preparation,* p* = 1.000, all other *p* < .001), familiarity (but not different from Appliances, *p* = 1.000, all other *p* < .001), appetizing (all *p < *.001), associated to sweet (all *p < *.001) and savory (but not different from Cooking/Preparation, *p* = 1.000, all other *p* ≤ . 045), and arousal (but not different from Appliances, *p* = 1.000, all other *p* ≤ .008). Appliances obtained the highest intensity (but not different from Serving/Consumption, *p* = 1.000, all other *p* < .001) and confidence ratings (but not different from Serving/Consumption, *p* = 1.000, all other *p* < .001). Finally, sounds related to Cooking/Preparation were rated the most healthful (all *p ≤ *.003).

### Associations between dimensions

The Pearson correlations between subjective dimensions are presented in Table 5. Overall, the correlations between evaluative dimensions were significant and positive, with several indicating moderate to strong associations (*r > *.400, Evans, 1996). These included the correlations between valence and arousal (*r* = .644), valence and intensity (*r* = .519), and intensity and arousal (*r* = .797). The more familiar stimuli were associated with higher confidence in identification (*r* = .664) and higher ratings in valence (*r* = .490), arousal (*r* = .603), and intensity (*r* = 554). The more familiar sounds were also evaluated as healthier (*r* = .431) and more appetizing (*r* = .491). Sounds evaluated as healthier were more associated with valence (*r* = .668), intensity (*r* = .528), and arousal (*r* = .565), and were seen as more appetizing (*r* = 728) and more associated with both taste dimensions, sweet (*r* = .561) and savory (*r* = .549).

**Table 5:** Correlations between evaluative dimensions

	1.	2.	3.	4.	5.	6.	7.	8.
1. Valence	-							
2. Familiarity	.490^***^	-						
3. Intensity	.519^***^	.554^***^	-					
4. Healthfulness	.668^***^	.431^***^	.528^***^	-				
5. Appetizingness	.834^***^	.491^***^	.585^***^	.728^***^	-			
6. Associated with sweet	.503^***^	.221^***^	.393^***^	.561^***^	.643^***^	-		
7. Associated with savory	.382^***^	.124^**^	.346^***^	.549^***^	.540^***^	.712^***^	-	
8. Arousal	.644^***^	.603^***^	.797^***^	.565^***^	.691^***^	.414^***^	.347^***^	-
9. Confidence	.313^***^	.664^***^	.393^***^	.297^***^	.321^***^	.165^**^	.086	.388^***^

^***^ *p* < .001, ^**^ *p* < .050

Associations to sweet and savory tastes were strongly correlated (*r* = .712). Sweeter sounds were also evaluated as more positive (*r* = .503), appetizing (*r* = .643), and arousing (*r* = .414), whereas sounds more associated with the savory dimension were also deemed more appetizing (*r* = .540). Sounds evaluated as more appetizing were rated high in all other evaluative dimensions (*r* ≥ .321).

### Individual differences in subjective ratings

As shown in Table 6, no significant differences were observed between women and men in mean ratings across evaluative dimensions, all *p* ≥ .062.

**Table 6:** Evaluations (*M*, *SD*) for each dimension for the total sample, women, and men, and mean difference test results

	Overall (*N* = 332)		Women (*n* = 231)		Men (*n* = 99)		Difference test		
	*M*	*SD*	*M*	*SD*	*M*	*SD*	*t*	*df*	*p*
Valence	3.87	0.86	3.83	0.90	3.97	0.77	1.493	217.0	.137
Familiarity	4.92	0.78	4.93	0.81	4.92	0.73	−0.161	328.0	.872
Intensity	4.31	0.84	4.27	0.89	4.42	0.72	1.581	224.7	.115
Healthfulness	3.80	0.69	3.75	0.72	3.91	0.59	1.874	328.0	.062
Appetizingness	3.87	0.83	3.84	0.89	3.95	0.66	1.251	247.3	.212
Associated to sweet	2.98	0.82	2.94	0.84	3.08	0.77	1.465	328.0	.144
Associated to savory	3.02	0.82	3.00	0.84	3.08	0.77	0.77	328.0	.442
Arousal	4.23	0.89	4.21	0.94	4.26	0.74	0.499	231.9	.618
Confidence	4.65	0.88	4.60	0.88	4.78	0.86	1.745	328.0	.082

Still, we observed positive (although weak) associations between other individual characteristics and evaluative dimensions. Age was positively associated with valence (*r* = .200, *p* < .001), intensity (*r* = .188, *p* < .001), healthfulness (*r* = .153, *p* = .005), appetizingness (*r* = .194, *p* < .001), and arousal (*r* = .128, *p* = .020). Likewise, participants who reported higher interest in food and nutrition also provided higher valence (*r* = .130, *p* = .018), intensity (*r* = .131, *p* = .017), appetizingness (*r* = .126, *p* = .022), arousal (*r* = .110, *p* = .045), and confidence (*r* = .121, *p* = .028) ratings. Overall self-ratings of cooking skills were positively associated with familiarity (*r* = .126, *p* = .022), intensity (*r* = .125, *p* = .023), appetizingness (*r* = .140, *p* = .010), sweet (*r* = .134, *p* = .015) and savory (*r* = .167, *p* = .002) taste dimensions, and confidence in sound identification (*r* = .124, *p* = .024). Frequency of cooking was positively associated with intensity (*r* = .146, *p* = .008), healthfulness (*r* = .122, *p* = .027), savory (*r* = .175, *p* = .001), and arousal (*r* = .112, *p* = .042). Confidence in cooking skills was only positively associated with the savory taste dimension (*r* = .112, *p* = .042), whereas liking cooking was positively associated with most evaluative dimensions: valence (*r* = .113, *p* = .040), familiarity (*r* = .131, *p* = .017), healthfulness (*r* = .109, *p* = .046), appetizingness (*r* = .132, *p* = .016), association to savory (*r* = .124, *p* = .024), and confidence (*r* = .129, *p* = .019). In contrast, CSS did not correlate with any evaluative dimensions, all *p* ≥ .132.

## Discussion

There is more to food perception than what happens in the mouth. Although tastes and flavors make up an important motivation for consumption (Liem & Russell, 2019), eating is a behavior that encompasses all the senses (Spence & Piqueras-Fiszman, 2014). Although not always seen as such, the auditory modality is increasingly recognized as a relevant sensory modality for food perception and acceptance (Guedes, Garrido, et al., 2023; Spence, 2016; Spence et al., 2019; Zampini & Spence, 2010). At the same time, the interest in audition in the context of multisensory marketing creates a higher demand for reliable research with sound stimuli (Knöferle & Spence, 2021). This paper presents the first large-scale database of kitchen and food sounds (*N* = 180) for research purposes. Specifically, we developed and validated a comprehensive set of auditory stimuli to support future empirical studies in experimental research and applied domains (e.g., food science, consumer behavior). Moreover, we provide insights into the contribution of individual factors to the evaluation of sound stimuli, with important implications for more tailored methodological choices.

One main motivation for developing the Kitchen and Food Sounds (KFS) dataset was the scarcity of validated auditory stimuli for research. Compared with the visual modality, validated datasets of sounds are disproportionately less common for general stimulus categories (Gerdes et al., 2014; Yang et al., 2018), particularly in the food domain. Currently, food sounds are scattered over different databases (e.g., Bradley & Lang, 2007; Fan et al., 2017; Hocking et al., 2013; Yang et al., 2018), without an overarching conceptual framework to allow comparability across stimuli, and lack appropriate evaluative dimensions for the particular requirements of food research. To address this limitation, the present norming study included two sets of evaluative dimensions. To facilitate flexible use of the stimuli and their comparability with other sources, the items were rated in general affective dimensions that are common to different databases across stimulus categories and sensory modalities (e.g., valence, arousal; Blechert et al., 2019; Guedes, Prada, Garrido, et al., 2023; Rodrigues et al., 2018; Souza et al., 2021; Yang et al., 2018). In addition, dimensions of healthfulness, appetizingness, and associations with sweet and savory tastes were included to address the specific needs of experiments in food research. Importantly, these dimensions are also shared with other food-related databases, for instance, in the visual modality (e.g., Blechert et al., 2019; Charbonnier et al., 2016; Prada et al., 2017; Toet et al., 2019). We also assessed familiarity with the stimulus and asked participants to identify each sound, indicating their confidence level in such identification.

The analysis of the identification responses suggests that, overall, the sounds were correctly identified or, at least, participants could recognize the general sound category (e.g., “microwave beeping” and “kitchen appliances,” respectively). This was particularly true for the Appliances and Serving/Consumption categories, which obtained accurate identification response (level 1) means above 50%. In contrast, sounds resulting from the manipulation of food or beverage packaging obtained the lowest relative frequency of accurate responses. Still, all categories show a range of accurately identified sounds and others that were not. For instance, even for the Packaging category, some sounds were clearly identified (e.g., opening a can of soda or a bottle of beer, S91 and S79, respectively). Higher identification may occur for products (such as the above) where packaging sounds provide relevant cues regarding the products' quality or the hedonic value of the subsequent consumption experience (Almiron et al., 2021; Spence & Wang, 2015, 2017).

With some exceptions, the stimuli of the KFS database covered different levels (low, moderate, high) of the evaluative dimensions under analysis, indicating good adequacy to a broad range of research purposes. The correlation analysis showed a high degree of association between evaluative dimensions. Generally, all the dimensions were positively correlated, with several pairs of variables showing moderate to strong associations. The high correlations between the affective dimensions of valence and arousal suggest that the more positive stimuli were also the most arousing, which differs from other norming studies of auditory stimuli (e.g., Guedes, Prada, Garrido, et al., 2023; Yang et al., 2018). The more favorable evaluation of the more familiar stimuli follows one pattern of association extensively described in the literature and suggests that the tendency to develop more positive attitudes toward familiar stimuli extends to food sounds (Ali & Peynircioǧlu, 2010; Freitas et al., 2018; Madison & Schiölde, 2017; Pereira et al., 2011; Zajonc, 1968). Interestingly, associations with other “positive” attributes were also observed, for example, the tendency to evaluate more familiar sounds as healthier and more appetizing. It is worth noting that, in some cases, the sound categories might be critical for interpreting the correlation results. For example, the correlations between healthiness and the sweet and savory taste associations might be surprising, as one could expect higher sugar/salt content to be associated with lower healthiness. However, healthiness ratings were higher for sounds associated with foods (e.g., cooking/preparation compared with packaging), and these sounds were also more strongly linked with the two taste categories. Unsurprisingly, the sounds more associated with the two taste attributes were also evaluated as more appetizing and, in the case of the associations with the sweet taste, also as more pleasant, further reinforcing the high hedonic value of sweet taste sensations (Beauchamp, 2016; Ventura & Mennella, 2011; Zhou & Tse, 2022).

The sociodemographic characteristics of the sample had a limited influence on the stimuli evaluation. Still, some dimensions (namely, valence, intensity, healthfulness, appetite, and arousal) appeared to differ to some extent according to participants’ age. Moreover, some food and cooking-related variables (e.g., interest in food and nutrition, cooking frequency) also seem to be associated with different evaluative judgments. Therefore, participants’ proficiency in the kitchen could be one aspect to attend to when planning studies with food sounds. Although these data do not allow us to make inferences in this regard, we would advise special care when dealing with expert samples, such as chefs, cooks, baristas, or sommeliers.

### Limitations and future directions

Although the reported findings seem to support a broad applicability of the stimuli presented here, some limitations should be considered. First, some evaluative dimensions show low levels of variability at the extremes. For instance, stimuli with low familiarity or high association with sweet and savory tastes were uncommon. This may be because the majority of sounds refer to routine activities for most people (e.g., cooking, washing dishes), and that only a modest number of items had a clear association with specific foods. Indeed, unlike visual stimuli, only a limited number of foods have clear sonic signatures (e.g., popcorn popping). Therefore, those interested in the sounds produced by the interaction with foods (e.g., preparation, mastication) might find it more challenging to find unfamiliar or highly sweet or savory sounds in this category. As the results of the sound identification task also reveal, food sounds pose challenges regarding their identity as well (see also Vickers, 1980). Several stimuli in the database were rarely identified by participants (e.g., some packaging sounds). Importantly, this was observed even when our instructions emphasized that all the sounds presented were related to the eating context. Nevertheless, having a heterogeneous set regarding identification may be convenient for researchers interested in using ambiguous auditory stimuli. A critical test would include asking participants to identify the sounds in the absence of a context or include cues associated with different contexts. It is possible that some sounds are (relatively) context-independent, whereas the interpretation of others relies on the context (e.g., the sound of cutting bread might be interpreted differently if paired with the sound/image/scent of wood). Another potential limitation is the large proportion of items in the moderate valence level, which suggests a relative prevalence of affectively neutral stimuli in this database. Consequently, there is a narrower range of stimuli for those interested in testing the modulatory potential of highly appealing or pleasant sounds.

One important aspect of norming research concerns the generalizability of norming data. One often-cited dimension of interest concerns cultural variability (Prada et al., 2017). This is a reason for special concern with food-related stimuli, considering the different dietary practices and how food is prepared and consumed (e.g., utensils like chopsticks vs. cutlery) across different social and cultural groups. Indeed, some food pictures databases have been validated in different countries. For example, the *food-pics* database was initially validated by Blechert et al. (2014) with American and German participants, and then a stimuli subset was validated in Portugal (Prada et al., 2017) and France (Bonin et al., 2021). The same rationale may be applied to auditory stimuli. For instance, while in Western countries the sound of toasting with champagne (e.g., stimulus #159) might readily come to mind in association with celebrations and special events, some cultural and/or religious groups shy away from toasting with alcoholic beverages. Likewise, for identification tasks, the sound of biting puff pastry (e.g., S165) may lead to different responses depending on the geography (e.g., the crunch of a pastel de nata in Portugal, a croissant in France, or baklava in some Middle Eastern countries).

### Conclusions and implications

The KFS database is the first large set of sounds associated with food and eating. Across 180 stimuli, this dataset covers sounds of preparing, cooking, serving, and/or consuming foods and beverages, as well as sounds of packaging, kitchen utensils, and appliances. In this paper, we provide open access to the full norming data to support the use of these stimuli in future research, as well as supplementary information regarding their suitability to different participant profiles.

As it becomes increasingly apparent that eating is fundamentally a multisensory event, studying the different sensory contributions to food perception is more relevant than ever (Spence, 2015; Velasco & Obrist, 2021). Acknowledging the necessity of validated stimuli in the auditory modality, this database may be suitable for research in unimodal but also in multimodal approaches (e.g., in combination with visual stimuli; e.g., Blechert et al., 2019; Charbonnier et al., 2016; Prada et al., 2017; Toet et al., 2019) that best mimic realistic eating situations. Given its broad range of stimulus categories and evaluative dimensions, this dataset may also suit different research domains, from fundamental (e.g., cognitive psychology, basic sensory science) to more applied research (e.g., marketing, consumer science).

Several studies suggest that sounds may be cross-modally associated with taste and flavor dimensions, with relevant implications for how these attributes are perceived (Guedes, Garrido, et al., 2023; Rodríguez et al., 2023). Sonic influences have been shown to influence not only food perception (Bravo-Moncayo et al., 2020; Lin et al., 2019; Xu et al., 2019; Zampini & Spence, 2004, 2005) but also behavior (Kaiser et al., 2016; Mathiesen et al., 2020, 2022; Stroebele & de Castro, 2006) and food choice (North et al., 1997, 1999; Peng-Li et al., 2020, 2021). The marketing value of sounds seems to accompany this realization, with promising applications to the digital world (Petit et al., 2019). Recent research seems to support this view, for instance, with musical stimuli to nudge shopping choices (Damen et al., 2021), or packaging sounds to improve sensory expectations toward products in e-commerce settings (Rodríguez et al., 2021). While digital outlets appear to make very limited use of sound compared to offline stores, researchers are urged to advance new knowledge on how these sensory cues may work for different settings, product segments, or consumer profiles (Fiore & Kelly, 2007). In addition to commercial applications, validated food sounds may also serve to advance research on the multisensory contributions to better eating. While more evidence emerges on the potential use of musical cues in improving perception and acceptance of healthier foods (e.g.,Guedes, Prada, Lamy, et al., 2023; Swahn & Nilsen, 2023; Techawachirakul et al., 2022), further research is needed to understand the potential application of sounds, such as those of food preparation, serving, or consumption, in improving the hedonic value of healthier and/or more sustainable products.

## Data Availability

All data and materials are available at OSF.
